# Altered Vaginal Microbiota Composition Correlates With Human Papillomavirus and Mucosal Immune Responses in Women With Symptomatic Cervical Ectopy

**DOI:** 10.3389/fcimb.2022.884272

**Published:** 2022-05-17

**Authors:** Mariana López-Filloy, Flor J. Cortez, Tarik Gheit, Omar Cruz y Cruz, Fernando Cruz-Talonia, Monserrat Chávez-Torres, Cristina Arteaga-Gómez, Ismael Mancilla-Herrera, Juan J. Montesinos, Víctor Adrián Cortés-Morales, Cecilia Aguilar, Massimo Tommasino, Sandra Pinto-Cardoso, Leticia Rocha-Zavaleta

**Affiliations:** ^1^Departamento de Biología Molecular y Biotecnología, Instituto de Investigaciones Biomédicas, Universidad Nacional Autónoma de México (UNAM), Ciudad de México, Mexico; ^2^International Agency for Research on Cancer, Lyon, France; ^3^Clínica de Colposcopia Fundación “Cruz Talonia”, Ciudad de Mexico, Mexico; ^4^Centro de Investigación en Enfermedades Infecciosas, Instituto Nacional de Enfermedades Respiratorias Ismael Cosío Villegas, Ciudad de México, Mexico; ^5^Deparatamento de Oncología, Instituto Nacional de Perinatología Isidro Espinosa de los Reyes, Ciudad de México, Mexico; ^6^Departamento de Infectología e Inmunología, Instituto Nacional de Perinatología Isidro Espinosa de los Reyes, Ciudad de México, Mexico; ^7^Mesenchymal Stem Cells Laboratory, Oncology Research Unit, Oncology Hospital, National Medical Center, Instituto Mexicano del Seguro Social (IMSS), Mexico City, Mexico

**Keywords:** human papillomavirus, vaginal microbiota, mucosal immune responses, genital inflammation, cervicovaginal mucus, cervical ectopy

## Abstract

Cervical ectopy is a benign condition of the lower genital tract that is frequently detected in women of reproductive age. Although cervical ectopy is regarded as a physiological condition, some women experience symptoms such as leucorrhoea, persistent bleeding and recurrent vaginal infections that require medical intervention. Cervical ectopy has not been linked to cervical cancer, but it is thought to facilitate the acquisition of sexually transmitted diseases (STDs), like Human Papillomavirus (HPV) infection, as it provides a favorable microenvironment for virus infection and dissemination. We and others have described the presence of oncogenic HPV types in women with symptomatic cervical ectopy. The relevance of this finding and the impact of symptomatic cervical ectopy on the cervicovaginal microenvironment (vaginal microbiota, immune and inflammatory responses) are currently unknown. To shed some light into the interplay between HPV, the vaginal microbiota and mucosal immune and inflammatory responses in the context of this condition, we enrolled 156 women with symptomatic cervical ectopy and determined the presence of HPV using a type-specific multiplex genotyping assay. Overall, HPV was detected in 54.48% women, oncogenic HPV types were found in more than 90% of HPV-positive cases. The most prevalent HPV types were HPV16 (29.4%), HPV31 (21.17%) and HPV18 (15.29%). Next, we evaluated the vaginal microbial composition and diversity by 16S rDNA sequencing, and quantified levels of cytokines and chemokines by flow cytometry using bead-based multiplex assays in a sub-cohort of 63 women. IL-21 and CXCL9 were significantly upregulated in HPV-positive women (*p*=0.0002 and *p*=0.013, respectively). Women with symptomatic cervical ectopy and HPV infection had increased diversity (*p*<0.001), and their vaginal microbiota was enriched in bacterial vaginosis-associated anaerobes (*Sneathia*, *Shuttleworthia*, *Prevotella*, and *Atopobium*) and depleted in *Lactobacillus* spp. Furthermore, the vaginal microbiota of women with symptomatic cervical ectopy and HPV infection correlated with vaginal inflammation (IL-1β, rho=0.56, *p*=0.0004) and increased mucosal homeostatic response (IL-22, rho=0.60, *p*=0.0001). Taken together, our results suggest that HPV infection and dysbiotic vaginal communities could favor a vaginal microenvironment that might delay the recovery of the cervical epithelium in women with symptomatic cervical ectopy and favor STDs acquisition.

## Introduction

Over the last decades or so, female reproductive health has been at the forefront of research as strong evidence supports bidirectional relationships between the cervicovaginal microenvironment and cervical cancer, viral acquisition and persistence, gynecologic and obstetric diseases and other benign conditions like cervical ectopy (Laniewski et al., 2020). During adolescence, pregnancy, and in women who take hormonal contraceptives, eversion of the endocervix exposes the columnar epithelium to the vaginal environment, forming a cervical ectopy (Jacobson et al., 2000; Reich and Fritsch, 2014). Cervical ectopy tissue is eventually replaced by newly-formed squamous epithelium through an irreversible physiological process of squamous metaplasia (Jacobson et al., 2000). Cervical ectopy is generally considered a benign condition, however, there is evidence linking cervical ectopy to an increased risk of acquisition of sexually transmitted diseases (STDs), like *Chlamydia trachomatis* (Lee et al., 2006), Human Immunodeficiency Virus (HIV) (Myer et al., 2006), and Human Papillomavirus (HPV) (Monroy et al., 2010). In a previous work, we reported that a high proportion of women with cervical ectopy, who went on to receive treatment, were positive for the presence of high-risk HPV types (HR-HPV) (Monroy et al., 2010). Persistent infection with HR-HPV has been associated with cervical cancer (Walboomers et al., 1999). The clinical relevance of HPV infection in women with cervical ectopy remains unclear as cervical ectopy is not considered a malignant condition. Also, a number of women with cervical ectopy suffer from bothersome symptoms like abundant leucorrhoea, postcoital and intermenstrual bleeding, pelvic pain and recurrent cervicitis, that can impact their quality of life (Harry et al., 2007; Hua et al., 2012; Cekmez et al., 2016). The presence of chronic symptoms, along with the risk of further infections, warrants treatment of cervical ectopy, mainly cauterization of the area (Machado et al., 2008).

The natural history of cervical ectopy is influenced by the vaginal microenvironment and modified by the presence of reproductive hormones (estrogen), trauma (sexual activity), and the pH of the cervicovaginal mucus. Indeed, squamous metaplastic activity is stimulated by the acidic pH of the vagina (Jacobson et al., 2000), which is in turn regulated by the vaginal microbiota. Low vaginal pH (< 4.5) is maintained by the activity of most species of *Lactobacillus* (*L. crispatus*, *L. iners*, *L. jensenii*, and *L. gasseri*), which colonize the cervicovaginal mucus (Lamont et al., 2011) and constitute what is considered an optimal vaginal microbiota. The interaction between epithelial cells, cervicovaginal mucus and vaginal microbiota, is important to preserve female reproductive health, by providing a biological barrier that prevents acquisition of STDs (Amabebe and Anumba, 2018), among other functions. Accordingly, recent studies have demonstrated that infection of the squamous epithelium with HR-HPV was associated with a vaginal microbiota dominated by bacterial vaginosis-associated bacteria (Wei et al., 2021) and depleted in *Lactobacillus* spp. (Andrade Pessoa Morales et al., 2021). Moreover, HPV-associated precancerous cervical lesions are also linked with changes in the vaginal microbial composition (Nieves-Ramírez et al., 2021). Very little is known about the vaginal microbial ecology in women with symptomatic cervical ectopy, as a result, the potential interactions between the vaginal microbiota and HPV infection are also currently unknown. This is possibly related to the fact that cervical ectopy is considered a common condition that is often overlooked and understudied.

A general feature of women with symptomatic cervical ectopy is the abnormal discharge of cervicovaginal mucus, which is produced by the everted glandular cells. Cervicovaginal mucus contributes to mucosal immune responses, because it harbors both cytokines and antibodies which are mediators of innate and adaptive immunity. Genital inflammation, often determined by levels of pro-inflammatory cytokines in cervicovaginal mucus, has been extensively studied in women with HPV-associated squamous lesions and tumors (Iwata et al., 2015; Daniilidis et al., 2016; Otani et al., 2019), but lesser so in women with cervical ectopy (Hwang et al., 2011; Kyongo et al., 2012). Our group has shown the presence of mucosal IgA anti-HPV antibodies in a proportion of HPV-infected women with cervical ectopy (Monroy et al., 2010). Although cervical ectopy has been associated with increased levels of pro-inflammatory cytokines, the potential impact of HPV infection in the development of mucosal innate and inflammatory responses in women with symptomatic cervical ectopy has not been fully explored. Moreover, local immune responses are thought to be regulated by the vaginal microbiota (Anahtar et al., 2018). For instance, the presence of Gram-negative bacteria in a Lactobacilli-enriched genital tract induces a Th17 response and the production of IL-23 (Witkin et al., 2011). To our knowledge, the impact of the vaginal microbiota in regulating inflammatory and cellular immune responses to HPV in women with cervical ectopy is unknown.

Thus, in the present work we determined the prevalence of HPV genotypes in glandular tissue of women with symptomatic cervical ectopy. Mucosal immune responses were evaluated in cervicovaginal mucus of a selected group of HPV-positive and HPV-negative women, in order to determine their genital inflammation profile (cytokines and chemokines). Next, we also characterized the vaginal microbiota by 16S rRNA sequencing. Finally, associations between HPV infection, vaginal microbial communities and genital inflammation were explored. Our study aims at improving our understanding of the complex interactions of distinct elements within the cervicovaginal environment of women suffering from a common, but scarcely explored condition of the female genital tract.

## Materials and Methods

### Study Population

Female participants were selected from women attending the Colposcopy Clinic of the “Cruz-Talonia” Foundation, Mexico City, Mexico, a non-profit institution dedicated to preventing cervical cancer, between 2017 and 2018. Women who complained of abnormal vaginal discharge, intermenstrual and/or post-coital bleeding were examined and underwent colposcopy. Women with symptomatic cervical ectopy, defined as irregular patches of columnar epithelium with papillae and clefts, without ectocervical changes after acetic acid application, were invited to participate. Eligible women: older than 20 years of age, sexually active, not pregnant, without a previous history of HPV-associated pathologies, and HIV seronegative, were invited to participate. All participants agreed to participate in this study, signed an informed consent form and answered a validated questionnaire on sociodemographic characteristics, sexual behavior, and medical history. This study was approved by the Local Ethics Committee of the Colposcopy Clinic of the “Cruz-Talonia” Foundation (Reference Number: 17/02/2017/LRZ/CE05). All women consented to provide a tissue biopsy and a cervicovaginal mucus sample, and to give any remaining unwanted samples to research.

### Sample Collection

All samples were obtained from participants at the seventh day of their menstrual cycle. Colposcopy-directed biopsies were obtained by trained specialists (colposcopists) experienced in the management of lower genital tract pathologies. Biopsies were transported on ice and processed immediately. Tissue biopsies were divided into fragments. One fragment was used for histopathology analysis to corroborate colposcopy-directed diagnosis, tissue biopsies were fixed in formalin and embedded in paraffin, 4 µm sections were subsequently deparaffinized, rehydrated and stained with hematoxylin-eosin to be analyzed by experienced pathologists. The second fragment was used for DNA extraction. Cervicovaginal mucus was collected by washing the cervix with 1mL of sterile phosphate-buffered saline. Mucus samples were transported on ice and stored at -20°C until used.

### HPV Detection

DNA was isolated from cervical eversion biopsies using the QIAamp DNA kit (Qiagen GmbH, Hilden, Germany), according to the manufacturer´s instructions. The presence and genotyping of HPV was performed using the type-specific multiplex genotyping (E7-MPG) assay, which combines multiplex polymerase chain reaction and bead-based Luminex technology (Luminex Co. Austin, TX, USA), at the International Agency for Research on Cancer (IARC), Lyon, France, as described elsewhere (Clifford et al., 2016). The assay was designed to simultaneously detect 21 mucosal HPV genotypes (HPV6, 11, 16, 18, 26, 31, 33, 35, 39, 45, 51, 52, 53, 56, 58, 59, 66, 68, 70, 73, and 82), which have been classified as HR-HPV genotypes (HPV16, 18, 31, 33, 35, 39, 45, 51, 52, 56, 58, and 59), putative HR-HPV types (pHR-HPV) (HPV26, 53, 66, 68, 70, 73, 82), and not carcinogenic or low risk types (LR-HPV) (HPV6, and 11) (IARC, 2007). The assay includes oligonucleotides for the amplification of β–globin as a control for DNA integrity. Only samples that tested positive for the amplification of β–globin were included in the study.

### 16S rRNA Gene Sequencing: DNA Extraction, Amplicon Generation and Sequencing

A total of 63 samples were available for 16S sequencing. DNA was extracted from cervicovaginal mucus using the PureLink™ Microbiome DNA Purification Kit (ThermoFisher Scientific, Waltham, MA, USA). Libraries were prepared as described in the MiSeq 16S rRNA gene Amplicon Sequencing protocol (Illumina, San Diego, CA, USA) with modifications. Primers for the V4 region were 515F (5´- GTGCCAGCMGCCGCGGTAA-3´and 806R (5´- GGACTACHVGGGTWTCTAAT-3´) (Caporaso et al., 2011). Briefly, the V4 region was amplified by PCR (triplicate reactions of 25 µL per sample). Triplicate PCR amplifications were confirmed in a 2% agarose gel electrophoresis. Triplicate PCR reactions were pooled per sample and purified using AgenCourt AMPure XP beads (Beckman Dickson, Atlanta, GA, USA). Dual indices were attached using the Nextera XT Index Kit (Illumina). Next, indexed PCRs were purified twice using AgenCourt AMPure XP beads, and quantified using Qubit fluorometer (ThermoFisher Scientific, Waltham, MA, USA). Equimolar concentrations of indexed PCR libraries were pooled together at 4nM. Negative controls were also included to control for exogenous contaminants. Quality control and molarity of the final library was assessed on the Agilent® High Sensitivity DNA Kit/2100 Bioanalyzer (Agilent, Santa Clara, CA, USA). Sequencing was performed on an Illumina MiSeq™ platform (Illumina) according to the manufacturer’s specifications. The final library was sequenced at 8pM concentration with 25% PhiX to generate paired-end reads of 500 base-length (v2 2x250 cycles). A total of 13,115,362 sequences were obtained from a single run.

### 16S Data Analysis

Raw Illumina MiSeq sequences were processed using the Qiime2 (Quantitative Insights into Microbial Ecology, version 2019.4) (Bolyen et al., 2019). First, sequences that might arise from sequencing errors or low abundance were filtered out, chimeras were filtered out as well, then any PhiX reads were removed and construction of a feature table (selection of amplicon variant sequences (ASVs)) were performed using dada2 (Callahan et al., 2016), options were: –p-trim-left-f 19, –p-trim-left-r 20, –p-trunc-len-r 180 and –p-trunc-len-f 185. This method allows for distinct reads with as few as 1 nucleotide difference to be clustered into separate ASVs. A total number of 467 ASVs were identified, and a median of 177,565 (71,692-453,044) sequences were retained after dada2. Taxonomy was assigned to ASVs using the q2‐feature‐classifier using the classify‐sklearn naïve Bayes taxonomy classifier against the GreenGenes (13_8) 99% reference sequences and trained to the region of the target sequences, in our case for the V4 region (515F-806R) (McDonald et al., 2012). Phylogenetic tree was constructed with fasttree2 (*via* q2‐phylogeny) after aligning all ASVs with mafft (Katoh et al., 2002; Price et al., 2010). Artifacts generated with Qiime2 were imported into Phyloseq for further manipulation, and graph visualization (R V.3.6.2, The R Foundation for Statistical Computing (McMurdie and Holmes, 2013). Before continuing with further manipulation, rarefaction was performed at a sampling depth of 64,522 sequences/sample (corresponding to 90% of the sample with the least sequences). Principal Coordinate analyses (PCoA) were used to visualize microbial communities and permutational multivariate analysis of variance (PERMANOVA, Adonis function) was used to assess differences in beta diversity (default parameters: 999 permutations). We used 2 metrics for beta diversity (Bray-Curtis dissimilarity index and weighted UniFrac) (Lozupone et al., 2011). Linear discriminant analysis (LDA) effect size (LEfSe) was used to detect significant differentially abundances features (taxa) between groups (default parameters were used, LDA threshold= 3, alpha value for factorial Kruskal-Wallis test 0.05, alpha value for pairwise Wilcoxon test 0.05 (Segata et al., 2011). Spearman correlations were performed using rcorr() function and visualized using corrplot() function.

### Cytokines and Chemokines Determination

The following cytokines and chemokines were evaluated using bead-based multiplex immune-assays by flow cytometry according to the protocols of the manufacturer: Interleukin-1β (IL-1β), and Interleukin-12p70 (IL-12p70) (BD Cytometric Bead Array (CBA) Human Inflammatory Cytokines Kit. BD Biosciences, CA, USA); Interleukin-2 (IL-2), Interleukin-4 (IL-4), Interleukin-5 (IL-5), Interleukin-6 (IL-6), Interleukin-9 (IL-9), Interleukin-10 (IL-10), Interleukin-13 (IL-13), Interleukin-17A (IL-17A), Interleukin-17F (IL-17F), Interleukin-21 (IL-21), Interleukin-22 (IL-22), Interferon-γ (IFN-γ), and Tumor Necrosis Factor- α (TNF-α) (LEGENDplex™ Human Th Cytokine Panel (13-plex), BioLegend, CA, USA). MCP-1 (CCL2), RANTES (CCL5), IP-10 (CXCL10), Eotaxin (CCL11), TARC (CCL17), MIP-1α (CCL3), MIP-1β (CCL4), MIG (CXCL9), MIP-3α (CCL20), ENA-78 (CXCL5), GROα (CXCL1), I-TAC (CXCL11) and IL-8 (CXCL8) (LEGENDplex™ Human Proinflammatory Chemokine Panel. BioLegend, CA, USA). Briefly, cervicovaginal mucus samples were thawed and centrifuged at 3000 x *g* for 15 min at 4°C, supernatants were recovered and the total protein content was calculated using the DC Protein Assay (Bio-Rad Inc., CA, USA). Samples were analyzed in a FACS ARIA III flow cytometer (BD Biosciences, CA, USA). Cytokines and chemokines concentrations were calculated using the LEGENDplex™ Data Analysis Software v 7.0 (BioLegend, CA, USA). Cytokine and chemokine expression levels were normalized to total protein concentration. The lower limit of detection (LLOD) value, provided by the manufacturer, was assigned to those samples with cytokine or chemokine concentrations equal or below the reported limit. To control for inter-individual variation due to differences in the amount of protein contained in the samples, the final concentration of analytes was reported as pg/mg of total protein.

### Statistical Analysis

Data was analyzed using the GraphPad Prism 7 software (GraphPad, USA). Differences in demographic and clinical factors between groups were analyzed using the Mann-Whitney test for continuous variables and Chi-square test for categorical values. Comparisons between two groups were performed using the Wilcoxon Rank Sum Test. Comparisons between three or more groups were performed using Kruskal-Wallis, *p* values were adjusted for multiple comparisons using Dunn’s multiple comparisons test. When using this test, adjusted-*p* values were reported. Two-tailed *p* values (and adjusted *p* values for KW test) lesser than 0.05 were considered statistically significant.

## Results

### Prevalence of HPV in Women With Symptomatic Cervical Ectopy

**Figure 1:**
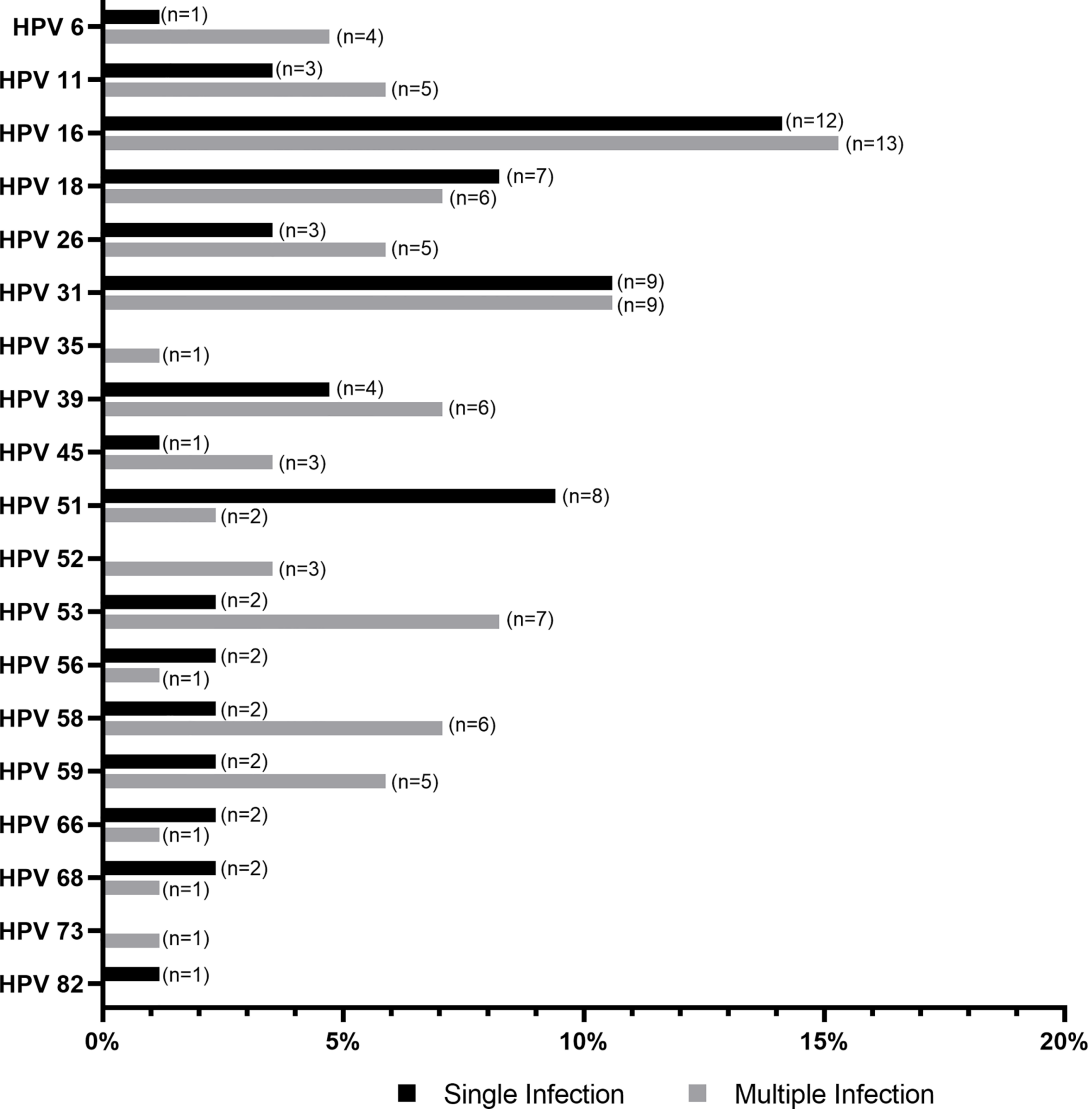
Histogram showing the distribution of human papillomavirus (HPV) genotypes detected as single and multiple infection, relative to the total number HPV-positive samples (N=85).

A total of 156 samples from women with symptomatic cervical ectopy were available for HPV testing. A total of 19 HPV types were detected: HPV6, 11, 16, 18, 26, 31, 35, 39, 45, 51, 52, 53, 56, 58, 59, 66, 68, 73, and 82. The prevalence and distribution of HPV genotypes is shown in **Table 1**. Overall, 85 (54.48%) women tested positive for HPV infection. Both single and multiple HPV infections were detected. A total of 59 women had a single HPV type with 93.22% testing positive for HR-HPV or pHR-HPV genotypes, and 6.77% for LR-HPV genotypes. HPV infection with multiple types accounted for 30.58% of all HPV types (26/85), and all women in this category tested positive for HR-HPV types. The relative frequency of HPV genotypes detected as single and multiple infections is shown in **Figure 1**. HPV16 was the most predominant, detected in 29.41% of the positive samples, followed by HPV31 (21.17%), and HPV18 (15.29%) (**Figure 1** and **Supplementary Table 1**). Women with HPV infection (HPV-pos) were younger than HPV negative (HPV-neg) women (*p*=0.0459, **Table 2** and **Supplementary Figure 1**). Of interest, although all women reported to be sexually active and had had children, a total of 140 women reported never having had a Papanicolaou (PAP) smear test, with no difference being observed between HPV-neg and HPV-pos women. A total of 17 women were vaccinated against HPV with the 4-valent HPV vaccine targeting HPV-6, -11, -16, and -18 types (Gardasil^®^, MSD), again no difference was observed between HPV-neg (8/17) and HPV-pos (9/17) women. It is worth mentioning that 3 of the HPV-vaccinated women were positive for HPV-16, whereas 6 HPV-vaccinated women were infected with HPV types not included in the vaccine. Taken the latter in consideration, and the prevalence of HR-HPV types in our cohort, these women might be at increased risk for HPV-associated pathologies.

**Table 1:** HPV prevalence and genotype distribution in women with symptomatic cervical ectopy.

HPV	Total (N=156)
Type	N (%)
Negative	71 (45.51)
Any HPV	85 (54.48)
High Risk	
16	25 (16.03)
18	13 (8.33)
31	18 (11.53)
35	1 (0.64)
39	10 (6.41)
45	4 (2.56)
51	10 (6.41)
52	3 (1.92)
56	3 (1.92)
58	8 (5.13)
59	7 (4.49)
Putative High Risk	
26	8 (5.13)
53	9 (5.77)
66	2 (2.56)
68	2 (1.28)
73	1 (0.64)
82	1 (0.64)
Low Risk	
6	5 (3.20)
11	8 (5.13)

HPV, Human Papillomavirus.

**Table 2:** Clinical, demographic and risk factors associated with HPV infection.

Characteristic	HPV-pos (N=85)	HPV-neg (N=71)	*p* value
Age (years)	30.77	32.75	0.0459^a*^
Parity	2.14	2.07	0.8949^a^
Menarche (years)	12.64	12.31	0.1612^a^
Age of sexual debut (years)	17.72	18.08	0.6256^a^
Number of sexual partners	2.21	1.82	0.0550^a^
Smoking status (%)			0.7196^b^
Current smoker Non-smoker	16 (18.8) 69 (81.2)	15 (21.1) 56 (78.9)	
Contraception (%)			0.4949^b^
None Physical^c^ Hormonal^d^ Chirurgical^e^	24 (28.2) 38 (44.7) 9 (10.6) 14 (16.5)	26 (36.6) 27 (38.0) 10 (14.1) 8 (11.3)	
HPV vaccine (%)			0.8921^b^
Yes No	9 (10.6) 76 (89.4)	8 (11.7) 63 (88.7)	
Prior pap smear (%)			0.7036^b^
Yes No	8 (9.4) 77 (90.6)	8 (11.7) 63 (88.7)	
Chronic diseases and conditions (%)			0.4781^b^
Arthritis Asthma Diabetes Hypertension Other	1 (1.2) 2 (2.4) 1 (1.2) 1 (1.2) 3 (3.5)	0 (0.0) 1 (1.4) 3 (4.2) 3 (4.2) 1 (1.4)	

Values are given as mean and number (%).

HPV, Human Papillomavirus.

HPV-pos, HPV positive.

HPV-neg, HPV negative.

a By Mann-Whitney test.

b By Chi-square test.

c Includes condoms, and intrauterine device (IUD).

d Includes implants, injections, patches, and pills.

e Tubal ligation surgery.

*p < 0.05 was considered significant.

Subsequent analyses were performed in a sub-cohort of 63 women including 35 HPV-pos and 28 HPV-neg women. This sub-cohort was selected based on sample availability (characteristics and HPV prevalence and genotype distribution are summarized in **Supplementary Tables 2**, **3** respectively).

### Symptomatic Cervical Ectopy Is Associated With Generalized Inflammation While HPV Infection Is Associated With High Levels of IL-21 and CXCL9

Next, we explored the impact of HPV infection on mucosal inflammation as it is well documented that persistent HPV infection is associated with increased levels of local and systemic inflammatory cytokines (Kemp et al., 2010; Scott et al., 2013). Sixteen pro-inflammatory cytokines and 12 chemokines were determined in cervicovaginal mucus. Levels of IL-8, IL-1β, IL-22, CCL17, and CCL3 were comparable between HPV-pos and HPV-neg women (**Table 3**). These 3 cytokines and 2 chemokines were detected in more than 90% of women suggesting that most women with symptomatic cervical ectopy suffered from an inflammatory response independent of the presence of HPV. As shown in **Table 3**, levels of IL-21 (27.74 pg/mg in HPV-pos *vs* 4.89 pg/mg in HPV-neg women) and MIG (56.89 pg/mg in HPV-pos vs 15.34 pg/mg in HPV-neg women) were significantly higher in HPV-pos women (*p*=0.0002 and *p*=0.013, respectively), which is suggestive of an anti-viral cellular immune response.

**Table 3:** Cytokine and chemokine concentrations in cervicovaginal mucus from women with symptomatic cervical ectopy.

Analyte	HPV-pos		HPV-neg		*p* value^a^
	N=35 (pg/mg)		N=28 (pg/mg)		
	Median	IQR	Median	IQR	
Cytokines					
IL-1β	693.10	(213.5-1574)	485.50	(313.9-1416)	0.833
IL-2	4.03	(2.3-9.6)	1.79	(0.7-3.9)	0.114
IL-4	5.44	(1.8-16.2)	1.95	(0.9-3.3)	0.145
IL-6	13.08	(5.9-66.4)	13.89	(5.5-87)	0.922
IL-8	6435.00	(2290-13743)	4591.00	(2957-8609)	0.226
IL-9	3.39	(2.3-7.4)	2.19	(1.5-3.3)	0.080
IL-12p	21.55	(16.9-26.2)	45.17	(35.2-83.6)	0.143
IL-13	10.47	(5.2-25.7)	9.80	(3.6-15.9)	0.857
IL-17B	4.24	(3.9-8.1)	1.60	(0.5-4.1)	0.143
IL-21	16.24	(3.5-30.1)	2.02	(1.7-5.6)	0.0002*
IL-22	983.60	(463.7-2402)	973.50	(348.6-1710)	0.518
TNF-α	26.20	(13.1-44)	17.45	(6.8-34.9)	0.413
IFN-γ	20.18	(3.9-50.7)	8.64	(3.3-16.7)	0.445
Chemokines					
CXCL10	18.06	(10.6-35.2)	22.61	(7.4-41.5)	0.682
CCL11	8.54	(6.2-11.8)	10.73	(4-26.5)	0.576
CCL17	9.97	(7.4-15.5)	9.68	(3.9-24.1)	0.715
CCL2	26.47	(9.7-85.6)	44.90	(20.4-85.1)	0.378
CCL5	19.82	(11.5-40.4)	18.71	(10.2-61.4)	0.865
CCL3	19.77	(10.2-28.3)	16.83	(7.7-39.7)	0.608
CXCL9	22.26	(8.7-76.9)	11.90	(4.2-21.1)	0.013*
CXCL5	53.24	(26.3-162.5)	26.46	(18-79.5)	0.069
CCL20	20.84	(10.4-37.7)	21.49	(6.4-49.2)	0.814
CXCL1	30.06	(18.5-77.1)	43.35	(12.6-75.4)	0.926
CXCL11	7.06	(3.8-16.9)	11.22	(3.1-19.2)	0.771
CCL4	22.83	(12.6-36.8)	23.06	(6.4-52.2)	0.684

Values are given as median (IQR 25-75).

HPV, Human Papillomavirus.

HPV-pos, HPV positive samples.

HPV-neg, HPV negative samples.

a By Mann-Whitney test.

*p < 0.05 was considered significant.

### Changes in Vaginal Microbiota Composition Are Associated With HPV Infection

**Figure 2:**
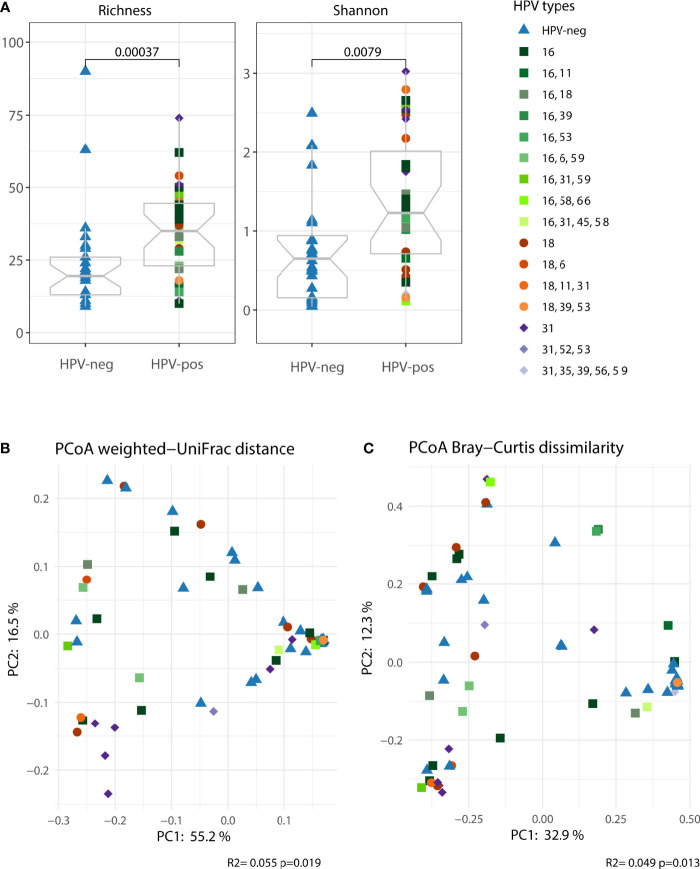
The vaginal microbiota (cervicovaginal mucus) was characterized in women with symptomatic cervical ectopy with or without HPV infection. **(A)** Two alpha diversity indexes were calculated, richness (observed species) and shannon. Boxplots depicting the median and interquartile range stratified by HPV infection. Groups were compared using the Wilcoxon Rank Sum test. Both weighted UniFrac **(B)** and Bray-Curtis dissimilarity **(C)** were used in conjunction with principal coordinate analysis (PCoA) to visualize microbial communities stratified by HPV infection. Differences in beta diversity were assessed using permutational multivariate analysis of variance (PERMANOVA). Each point was colored according to the HPV type for HPV-pos women and order by the number of HPV types found.

Our results suggest that women with symptomatic cervical ectopy suffer from a state of generalized mucosal inflammation which is not entirely explained by the presence of HPV. Strong evidence indicates that inflammatory responses in the female genital tract are modulated by the local microbiota (Anahtar et al., 2015). Thus, we investigated the microbiota composition in cervicovaginal mucus of women with symptomatic cervical ectopy. Both richness and shannon diversity were increased in HPV-pos compared to HPV-neg women (*p*<0.001, **Figure 2A**). Principal coordinates analysis (PCoA) was used to visualize the clustering of microbial communities stratified by HPV infection. HPV infection had an impact, albeit small, on the vaginal microbiota (explaining around 5% of variation, PERMANOVA *p*=0.019 for weighted UniFrac and *p*=0.013 for Bray-Curtis dissimilarity, **Figures 2B, C**, respectively). Given that HPV infection had a small albeit measurable impact on the vaginal microbiota, we next stratified HPV-positive women into two groups, those with single HPV infection (representing the leading 3 most prevalent HR-HPV types, HPV16, HPV18 and HPV31) and those with multiple HPV infection. Women with single HPV infection had significantly increased microbial richness and diversity (Richness, adjusted p=0.0021 and Shannon, adjusted p=0.0021. **Supplementary Figure 2A**) compared to HPV-negative women. Women with multiple HPV infection had increased richness (Richness, adjusted p=0.041) but not diversity (Shannon, adjusted p>0.05. **Supplementary Figure 2A**). PCoA was used to visualize the clustering of microbial communities of the 3 groups: HPV-negative, single HPV infection and multiple HPV infection (**Supplementary Figures 2B, C**). Single HPV infection had a significant impact on the vaginal microbiota (weighted UniFrac PERMANOVA *p*=0.014 and Bray-Curtis *p*=0.003). Multiple HPV infection had no impact on the vaginal microbiota (weighted UniFrac PERMANOVA *p*=0.149 and Bray-Curtis *p*=0.122). Results seem to indicate that differences in vaginal microbial composition between HPV-negative and HPV-positive women might be driven by single HPV infection, rather than multiple HPV infection. Therefore, next we restricted our analysis to the 3 predominant HR-HPV types found in our study. The prevalence of HPV16, HPV18 and HPV31 single infection was 8/35 (22.9%), 5/35 (14.3%) and 7/35 (20%), respectively.

**Figure 3:**
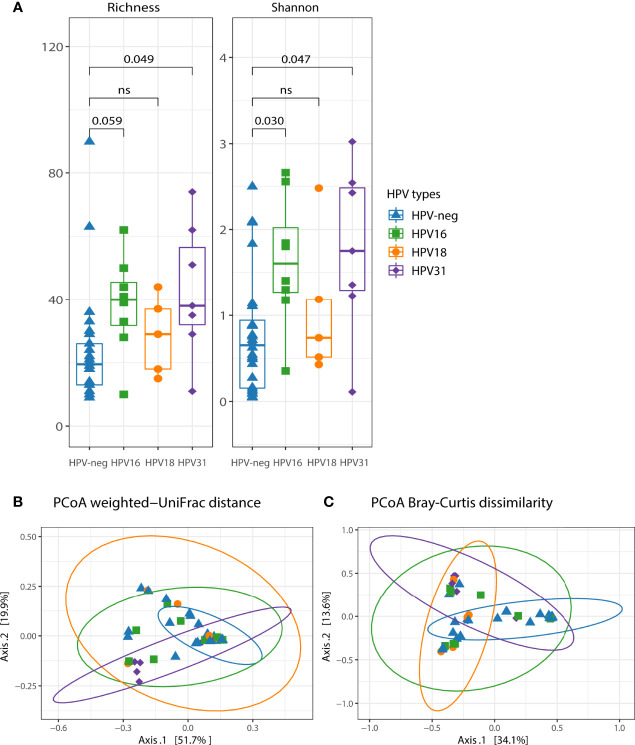
The impact of single HPV16, HPV31 and HPV18 on the vaginal microbiota diversity and community membership. **(A)** Two alpha diversity indexes were calculated, richness (observed species) and shannon. Boxplots depicting the median and interquartile range stratified by single HPV infection. Groups were compared using the Kruskal-Wallis test, *p* values were adjusted for multiple comparisons. Both weighted UniFrac **(B)** and Bray-Curtis dissimilarity **(C)** were used in conjunction with principal coordinate analysis (PCoA) to visualize microbial communities stratified by single HPV infection. Differences in beta diversity were assessed using permutational multivariate analysis of variance (PERMANOVA). Each point was colored according to the single HPV type. Confidence ellipses are shown for each group.

HPV16, HPV31, and HPV18 were the most prevalent genotypes in our cohort. Thus, we further analyzed the microbial composition including only women with single HPV16, single HPV31, and single HPV18 infection. Bacterial richness and diversity were increased in HPV16-positive (Richness, adjusted *p*=0.059; Shannon, adjusted *p*=0.030) and HPV31-positive (Richness, adjusted *p*=0.049; Shannon, adjusted *p*=0.047) compared to HPV-neg women (**Figure 3A**). We found that microbiota diversity was not significantly affected by the presence of HPV18 (*p*>0.1 for both alpha diversity indices) (**Figure 3A**). Beta diversity analyses showed bacterial community differences between HPV-neg women and HPV31-positive women (explaining 11.9% of variation, PERMANOVA *p*=0.004 for weighted UniFrac and 7% of variation, PERMANOVA *p*=0.012 for Bray-Curtis dissimilarity) (**Figures 3B, C**), and HPV18-positive women (PERMANOVA *p*=0.044 for weighted UniFrac, and PERMANOVA *p*=0.02 for Bray-Curtis dissimilarity) (**Figures 3B, C**). However, no differences in bacterial communities between HPV16-positive and HPV-neg women were observed (weighted UniFrac PERMANOVA *p*=0.114 and Bray-Curtis PERMANOVA *p*=0.061) (**Figures 3B, C**).

**Figure 4:**
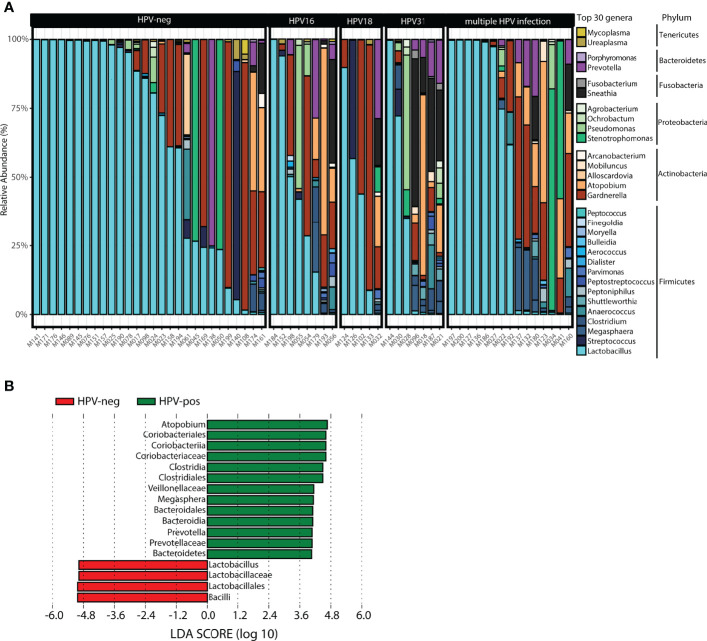
Taxonomic profile of the vaginal microbiota at genus level in women with symptomatic cervical ectopy. **(A)** Taxa barplots showing the taxonomic composition at genus level. The top 30 genera are represented, these account for 99.5% of all genera present. Genera are listed from the less abundant to the more abundant within each phylum, and ordered in ascending order (Tenericutes, Bacteroidetes, Fusobacteria, Proteobacteria, Actinobacteria, and Firmicutes). Color bars reflect the phyla they belong to and the intensity of the color their relative abundance, with light colors depicting the least abundant, and the darker colors the most abundant. **(B)** Histogram of the linear discriminant analysis scores reveals that 17 differential features were identified. *Atopobium*, *Megasphaera*, *and Prevotella* were found enriched in HPV-pos women while *Lactobacillus* was enriched in HPV-neg. Linear discriminant analysis Effect Size (LEfSe) analysis was performed with LDA values of 3.0 or higher.

The taxonomic profiling of the vaginal microbiota is shown in **Figure 4**. The top 30 genera stratified by HPV status, ordered by the relative abundance of *Lactobacillus* in each woman is shown in **Figure 4A**. The top three genera were *Lactobacillus*, *Gardnerella* and *Atopobium*. HPV31-positive women had increased *Sneathia*, *Shuttleworthia*, *Pseudomonas*, *Prevotella*, *Megasphaera*, *Clostridium*, *Atopobium*, and decreased *Lactobacillus* spp. and *Gardnerella* compared to HPV-neg women, whereas HPV-16 women showed increased *Sneathia, Pseudomonas, Megasphaera, Atopobium*, and decreased *Lactobacillus* spp. Although changes in microbiota diversity were not significant for HPV18-positive women, we observed a tendency for an increase in *Sneathia, Prevotella*, and *Gardnerella*, along with a decrease in *Lactobacillus* spp. (**Figure 4A**). LEfSe was used to detect significant differentially abundances features (taxa) between HPV-pos and HPV-neg women (LDA threshold of 3 or higher, other default parameters were used). A total of 17 features were identified. In particular, 3 genera were found to differentiate HPV-pos women (*Atopobium*, *Megasphaera, Prevotella*) (**Figure 4B**). In contrast, *Lactobacillus* was identified as a discriminant feature in HPV-neg women (**Figure 4B**). No discriminant features were found when analysing single vs multiple HPV infections or single HPV16, HPV31 and HPV18 infections.

### Associations Between Pro-Inflammatory Cytokines and Vaginal Microbiota in the Context of HPV Infection

Finally, we explored associations between genital inflammation and vaginal microbiota (richness, diversity and top 30 genera) in the context of HPV infection. We restricted our analysis to the cytokines and chemokines who were determined in 85% or more samples (IL-1β, IL-8, IL-22, CCL11, CCL17, CCL2, CCL5, CCL3, CXCL5, and CXCL1) (**Supplementary Table 4**). When the entire cohort was considered, irrespective of HPV status, we found strong inverse correlations (rho >-0.60, *p*<0.001) between *Lactobacillus* spp., bacterial-vaginosis associated anaerobes and vaginal richness and diversity, as would be expected (**Supplementary Figure 3**). At the same time, positive correlations (rho>0.40, *p*<0.001) were found between alpha diversity and most anaerobes. No associations were found between inflammation-associated cytokines and chemokines and the vaginal microbiota.

**Figure 5:**
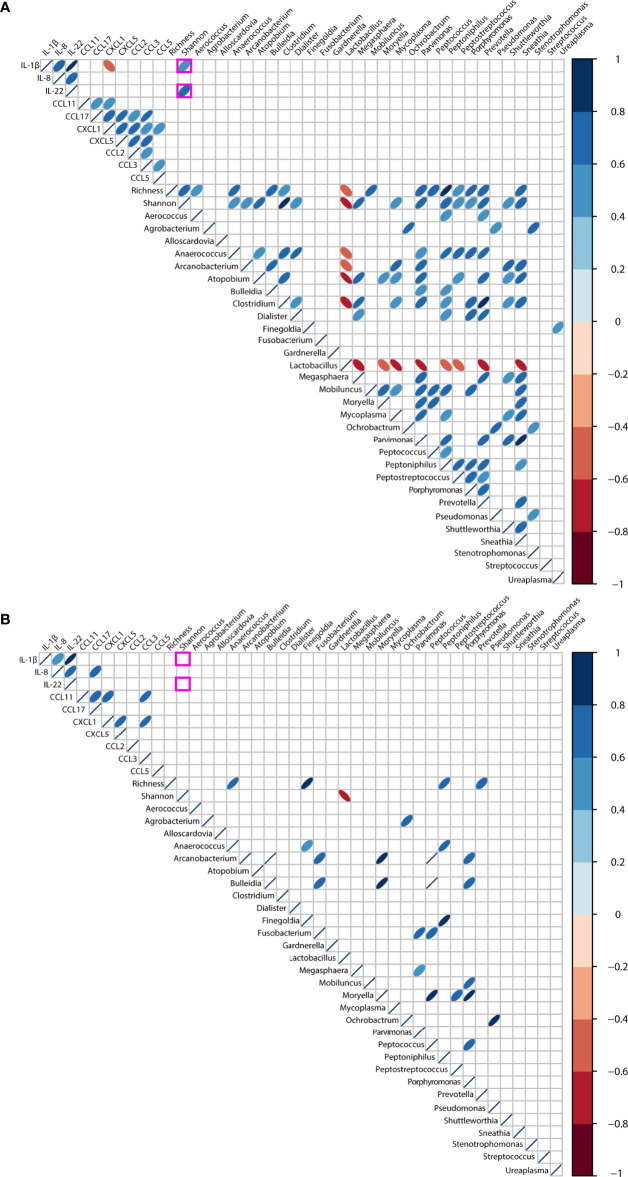
Correlations between the vaginal microbiota and genital inflammation in women with symptomatic cervical ectopy with **(A)** or without **(B)** HPV infection. Correlations were computed using Spearman test, and visualized using corrplot. Only *p* values< 0.01 are shown. Positive rho values are shown in blue, negative rho values shown in red, the intensity of the color is proportional to the strength of the rho value.

When stratifying for HPV infection, distinct correlations were found. In HPV-pos women, we found positive correlations between alpha diversity and IL-1β (rho= 0.56, *p*=0.0004) and IL-22 (rho= 0.60, p=0.0001) as shown in **Figure 5A**. Negative correlations (rho>-0.60, *p*<0.001) were found between *Lactobacillus* spp. and alpha diversity, and anaerobes like *Atopobium*, *Clostridium*, *Megasphaera*, *Mycoplasma*, *Prevotella*, and *Sneathia*. In contrast, no correlations were observed between richness, diversity and inflammation-associated cytokines and chemokines in HPV-neg women (**Figure 5B**). At the same time, positive correlations (rho>0.40, *p*<0.001) were found between alpha diversity indices and most anaerobes in both HPV-pos and HPV-neg women, however fewer correlations were found compared to HPV-pos women.

## Discussion

Here, we present data on the prevalence of HPV infection and distribution of HR-HPV types, the presence and magnitude of genital inflammation in cervicovaginal mucus, the diversity and taxonomic composition of the vaginal microbiota, and associations in women with symptomatic cervical ectopy, a common gynaecological condition, associated with hormonal changes, that either resolves by itself or requires treatment when symptoms (bleeding and recurrent vaginal infections) persist.

The presence of cervical ectopy has been associated with an increased risk of sexually transmitted infections (Lee et al., 2006; Monroy et al., 2010). Here, we detected the presence of HPV in more than half of women with symptomatic cervical ectopy, in particular in younger women (20-30 years of age). Our results are comparable to other studies (Nielsen et al., 2008; Bruni et al., 2010). We found high prevalence of single and multiple HR-HPV and pHR-HPV types, including HPV16, HPV31 and HPV18. We and others have previously reported the presence of oncogenic HPV types in women with cervical ectopy (Monroy et al., 2010; Hwang et al., 2011), however, since endocervical cells are unable to sustain productive HPV life cycle, the clinical relevance and implications of HR-HPV infection in everted endocervical epithelium remain controversial. Interestingly, a refined model of cervical carcinogenesis, that considers the participation of endocervical cells as potential precursors of cervical cancer has been proposed (Doorbar and Griffin, 2019). In line with this thinking, cervical ectopy represents a challenge, because in this condition endocervical cells are exposed to the complex vaginal microenvironment containing chemical, immune and biological elements that may alter the natural evolution of columnar epithelium to metaplastic squamous tissue.

In women with cervical ectopy, endocervical cells protruding onto the ectocervix create an additional mucosal barrier with a natural vulnerability to physical trauma that may lead to increased genital inflammation in sexually active women (Kyongo et al., 2012). Accordingly, we found the co-expression of five important mediators of inflammation, IL-1β, IL-22, IL-8, CCL17, and CCL3 in the vast majority of women included in this study. Our results are consistent with previous reports showing increased levels of cervicovaginal pro-inflammatory cytokines in healthy women with cervical ectopy (Hwang et al., 2011; Kyongo et al., 2012). However, the impact of HPV infection on the inflammatory responses in women with symptomatic cervical ectopy has not been previously evaluated. We could not detect differences in pro-inflammatory cytokines between HPV-pos and HPV-neg women, suggesting that HPV infection does not increase inflammation further. Our results agree with Kriek and colleagues (Kriek et al., 2016), who did not find an association between pro-inflammatory cytokines and HPV in endocervical samples. In contrast, a pro-inflammatory microenvironment has been identified in squamous epithelium persistently infected by HPV, where E5, E6 and E7 oncoproteins were found to be the main activator of inflammation-associated pathways (Hemmat and Bannazadeh, 2019). Glandular cells are unable to sustain HPV replication cycle, and expression of oncoproteins. This might explain why the presence of HPV is not associated with increased genital inflammation in our cohort.

HPV is one of the most common pathogens affecting the cervical canal. Cellular immune responses are decisive for the resolution of HPV infections (Stanley, 2006). Here, we detected increased levels of IL-21 and CXCL9 in HPV-pos women. IL-21 is a crucial factor for the immunological control of viral infections. IL-21 is produced by CD4+ T lymphocytes and natural killer T (NKT) cells, and it modulates the anti-viral activities of cytotoxic T lymphocytes (CTL) and natural killer (NK) cells (Spolski and Leonard, 2008). IL-21 may contribute to both initial immune responses and control of HPV-associated genital warts (Abu El-Hamd et al., 2019). Thus, it is reasonable to assume that elevated concentrations of IL-21 detected in our cohort, might reflect a cellular immune response against HPV. As already stated, IL-21 is produced by NKT cells. Migration of NKT cells to sites of infection and inflammation is mediated by chemokines such as CXCL9 (Ding et al., 2016). We detected increased levels of CXCL9 in HPV-pos women, which may be stimulating the recruitment of IL-21-producing NKT cells. An association of HPV infection with elevated levels of cervicovaginal CXCL9 was also reported by Shannon and colleagues (Shannon et al., 2017), however, gynecological conditions of women included in that study were not reported. Interestingly, the expression of CXCL9 can also be induced by bacterial lipopolysaccharide (LPS) (Bandow et al., 2012), suggesting that some bacteria within the vaginal microbiota may also influence the expression of chemokines and as a consequence the recruitment of cells to this site.

As shown by others, bacterial communities in the vagina are able to influence host mucosal immune responses (Agostinis et al., 2019). A vaginal microbiota is considered optimal if dominated by *Lactobacillus* species and a low microbial diversity. We found that the vaginal microbiota of HPV-pos women was highly diverse and differed in community composition (beta diversity) with a decrease in *Lactobacillus* dominance compared to HPV-neg women. We also found a strong correlation between alpha diversity metrics and IL-1β and IL-22, both of which are produced at sites of inflammation. These results are in line with a previous study showing correlations between highly diverse bacterial communities and genital pro-inflammatory cytokines (Anahtar et al., 2015). Also, changes in vaginal microbial composition have been shown to associate with female genital tract conditions including pelvic inflammatory disease (Ness et al., 2005). To our knowledge, this is the first report showing associations between specific pro-inflammatory cytokines, and increased bacterial diversity in women with symptomatic cervical ectopy in the context of HPV infection. Our results show that high bacterial diversity correlates with both IL-1β and IL-22. Also, NKT-recruiting factor CXCL9 was increased in the cervical mucus of HPV-pos women. Both results are of great interest to us. IL-1β induces the production of IL-22 by NKT cells as a response to bacterial invasion (Doisne et al., 2011). Likewise, levels of IL-22 are increased in women with HPV-associated genital warts (Radwa et al., 2021), thus it is likely that both, the altered bacterial communities and the presence of HPV may contribute to the elevated levels of IL-22 detected in our cohort.

The link between vaginal dysbiosis and HPV infection, persistence, premalignant lesions (Laniewski et al., 2018; Usyk et al., 2020), and ultimately cervical cancer has been clearly established (Brusselaers et al., 2019; Xie et al., 2020). Given that cervical ectopy is not considered a malignant condition, the potential association between HPV and vaginal dysbiosis in women with this condition remains unclear. As stated previously, a *Lactobacillus*-dominant is considered an optimal vaginal microbiota, whereas the presence of strict anaerobes is considered a state of dysbiosis (Pramanick et al., 2022). In our cohort, HPV-neg women were mostly *Lactobacillus* dominant, whereas HPV-pos women showed a significant decreased in Lactobacilli and increase in anaerobes such as *Sneathia, Pseudomonas, Megasphaera, Atopobium*, *Shuttleworthia*, *Prevotella*, and *Clostridium*. The presence of anaerobes is associated with high rates of bacterial vaginosis recurrence (Onderdonk et al., 2016) and cervicovaginal inflammation (Mitchell and Marrazzo, 2014). Furthermore, byproducts of dysbiotic bacteria metabolism may cause degradation of the mucosal surfaces of the glandular epithelium (Africa et al., 2014), and increase the vaginal pH in a number of women (Donders et al., 2016). In contrast, most *Lactobacillus* spp. are known to produce lactic acid and favor a low pH in the vagina. Importantly, low pH (pH 4.5) is a critical factor for the natural replacement of ectopic tissue with metaplastic squamous epithelium. Thus, the loss or decrease in the relative abundance of Lactobacilli may delay the recovery of the external stratified epithelium in women with cervical ectopy, possibly increasing their risk of acquiring STDs, including HPV and HIV.

Our results should be interpreted with caution due to the limitations in statistical power inherent to small cohort sizes like ours. We also acknowledge that we did not adjust for confounding variables; when comparing clinical and demographic variables between these two groups, we found that the number of sexual partners was significantly higher in HPV-positive women as previously reported. All other variables were not significant, with the exception of age. There was a tendency for HPV-negative women to be older than HPV-positive women.

We are aware that our study has important limitations; first, the inclusion of a control group of women without cervical ectopy was not possible, so we cannot compare our results with those of age-matched women without cervical ectopy. However, previous reports show that cytokines, chemokines and vaginal microbial community profiles are different in women with cervical ectopy compared with women with a normal ectocervix (Hwang et al., 2011; Kyongo et al., 2012). Taking this latter in consideration, we hypothesize that our results might have been in line with those previously published. Another limitation is the conceivable presence of HPV-infected squamous cells, in the biopsy used for HPV DNA determination. Although we performed an inspection of the cervix after acetic acid application, and we separated women showing an acetowhite lesion, we cannot exclude the possibility of an ongoing exocervical HPV infection with no clinical manifestations in some women. We are aware that recovering pure glandular tissue from biopsies using microdissection would be the ideal methodology. The cross-sectional nature of our study prevents us from proposing any associated mechanisms or causative relationships. To this end, longitudinal studies are in process. Finally, we are aware of the difficulty of classifying to species level with this approach (16S), so we only reported taxonomic profiles at the genus level.

In conclusion, we showed the presence of oncogenic HPV types in a high proportion of women with symptomatic cervical ectopy. Notably, HPV was associated with increased levels of IL-21 and CXCL9 suggesting an active cellular immune response. We also found the co-expression of inflammation markers in these women irrespective of HPV status. We identified that changes in vaginal microbiota composition were associated with the presence of HPV. *Lactobacillus* dominance decreased in HPV-pos women, and bacterial vaginosis-associated anaerobes were increased. Correlations between inflammation-associated cytokines IL-1β and IL-22 and the vaginal microbiota were exclusively found in HPV-pos women. Taken together, our observations suggest that HR-HPV infection and vaginal dysbiosis coexist in women with symptomatic cervical ectopy. These women might be more susceptible to chronic inflammation and epithelial damage, and could benefit from a closer follow-up to improve their sexual and reproductive health. Our study provides a deeper understanding of the cervicovaginal microenvironment of women with symptomatic cervical ectopy, a benign gynecological condition.

## Data Availability Statement

The raw 16S sequences have been deposited at the National Center for Biotechnology Information (NCBI)-Sequence Read Archive (SRA) under project PRJNA763215.

## Ethics Statement

The studies involving human participants were reviewed and approved by Local Ethics Committee of the Colposcopy Clinic of the “Cruz-Talonia” Foundation (Reference Number: 17/02/2017/LRZ/CE05). All women gave written informed consent to participate in this study.

## Author Contributions

LR-Z conceived the project, designed experiments, coordinated study operation, supervised laboratory staff, analyzed and discussed data, and wrote the manuscript. SP-C conducted 16S rRNA gene sequencing analysis, designed figures, analyzed and discussed data, and wrote the manuscript. ML-F performed flow cytometry analysis, sequencing reactions, analyzed data, designed figures and wrote the manuscript. FC generated clinical data base, processed tissue samples, extracted and analyzed DNA, and designed tables. TG conducted the E7-MPG assay to determine HPV genotypes. OC and FC-T coordinated study operation ant the Colposcopy Clinic of the “Cruz-Talonia” Foundation, and obtained tissue samples. MC-T extracted DNA from cervicovaginal mucus, constructed bacterial libraries, and performed 16S rRNA sequencing. CA-G analyzed cases and contributed to diagnosis confirmation. IM-H performed flow cytometry for chemokine determination. JM and VC-M performed flow cytometry analysis of cytokines. CA participated in the selection of patients, collection of patient data and processing of patient samples. MT coordinated study operation at the International Agency for Research in Cancer, analyzed and discussed data. All authors contributed to the article and approved the submitted version.

## Funding

This work was supported by a grant from PAPIIT, UNAM (grant number IN204419). LR-Z received a fellowship from Programa de Apoyos para la Superación del Personal Académico de la UNAM (PASPA. Number 523.01/2299DFA/2017). Grants from CONACyT (grant numbers 620702, 464745) were awarded to FC. SP-C was supported by funds from the Mexican Government (Programa Presupuestal P016, Anexo 13 del Decreto del Presupuesto de Egresos de la Federación).

## Author Disclaimer

Where authors are identified as personnel of the International Agency for Research on Cancer/World Health Organization, the authors alone are responsible for the views expressed in this article and they do not necessarily represent the decisions, policy or views of the International Agency for Research on Cancer/World Health Organization.

## Conflict of Interest

The authors declare that the research was conducted in the absence of any commercial or financial relationships that could be construed as a potential conflict of interest.

## Publisher’s Note

All claims expressed in this article are solely those of the authors and do not necessarily represent those of their affiliated organizations, or those of the publisher, the editors and the reviewers. Any product that may be evaluated in this article, or claim that may be made by its manufacturer, is not guaranteed or endorsed by the publisher.
