# Supplementary material for: Altered Vaginal Microbiota Composition Correlates With Human Papillomavirus and Mucosal Immune Responses in Women With Symptomatic Cervical Ectopy
Source: Front Cell Infect Microbiol. 2022 May 17;12:884272. doi: 10.3389/fcimb.2022.884272 (PMC9152460; doi:10.3389/fcimb.2022.884272)
Supplement: Supplementary file 2 [file Image_2.pdf]

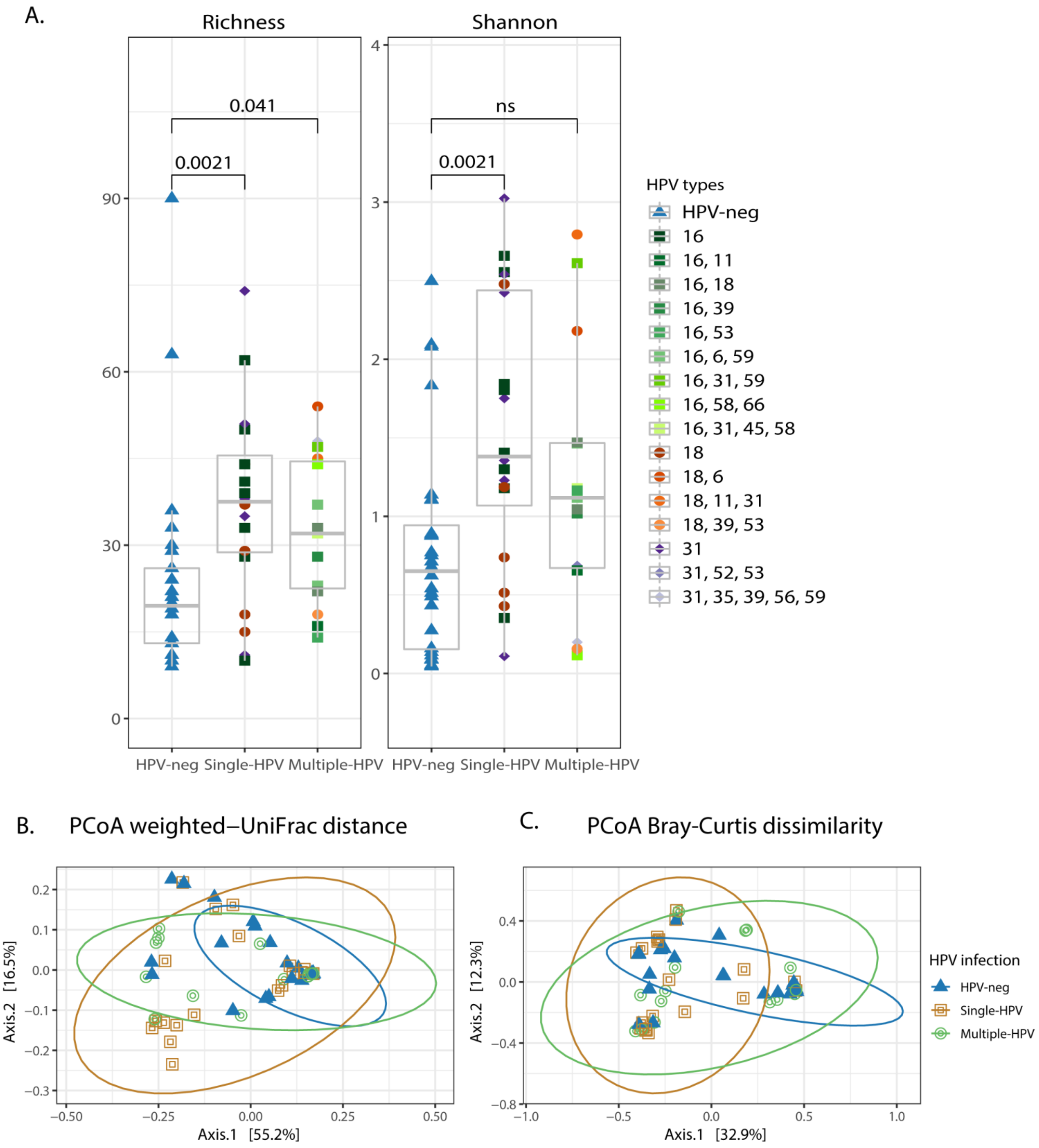

Supplementary Figure 2. The vaginal microbiota (cervicovaginal mucus) was characterized in women with symptomatic cervical ectopy with single and multiple HPV infection, or without HPV infection. **(A)** Two alpha diversity indexes were calculated, richness (observed species) and shannon. Boxplots depicting the median and interquartile range stratified by HPV infection. Groups were compared using the Kruskal-Wallis test. *p* values were adjusted for multiple comparisons using Dunn´s multiple comparisons test. Adjusted p values are shown. Both weighted UniFrac **(B)** and

Bray-Curtis dissimilarity **(C)** were used in conjunction with principal coordinate analysis (PCoA) to visualize microbial communities stratified by HPV infection (single vs multiple). Confidence ellipses are shown for each group. Differences in beta diversity were assessed using permutational multivariate analysis of variance (PERMANOVA). Each point was colored according to the HPV type for HPV-pos women and order by the number of HPV types found.
