# Supplementary material for: Altered Vaginal Microbiota Composition Correlates With Human Papillomavirus and Mucosal Immune Responses in Women With Symptomatic Cervical Ectopy
Source: Front Cell Infect Microbiol. 2022 May 17;12:884272. doi: 10.3389/fcimb.2022.884272 (PMC9152460; doi:10.3389/fcimb.2022.884272)
Supplement: Supplementary file 3 [file Image_3.pdf]

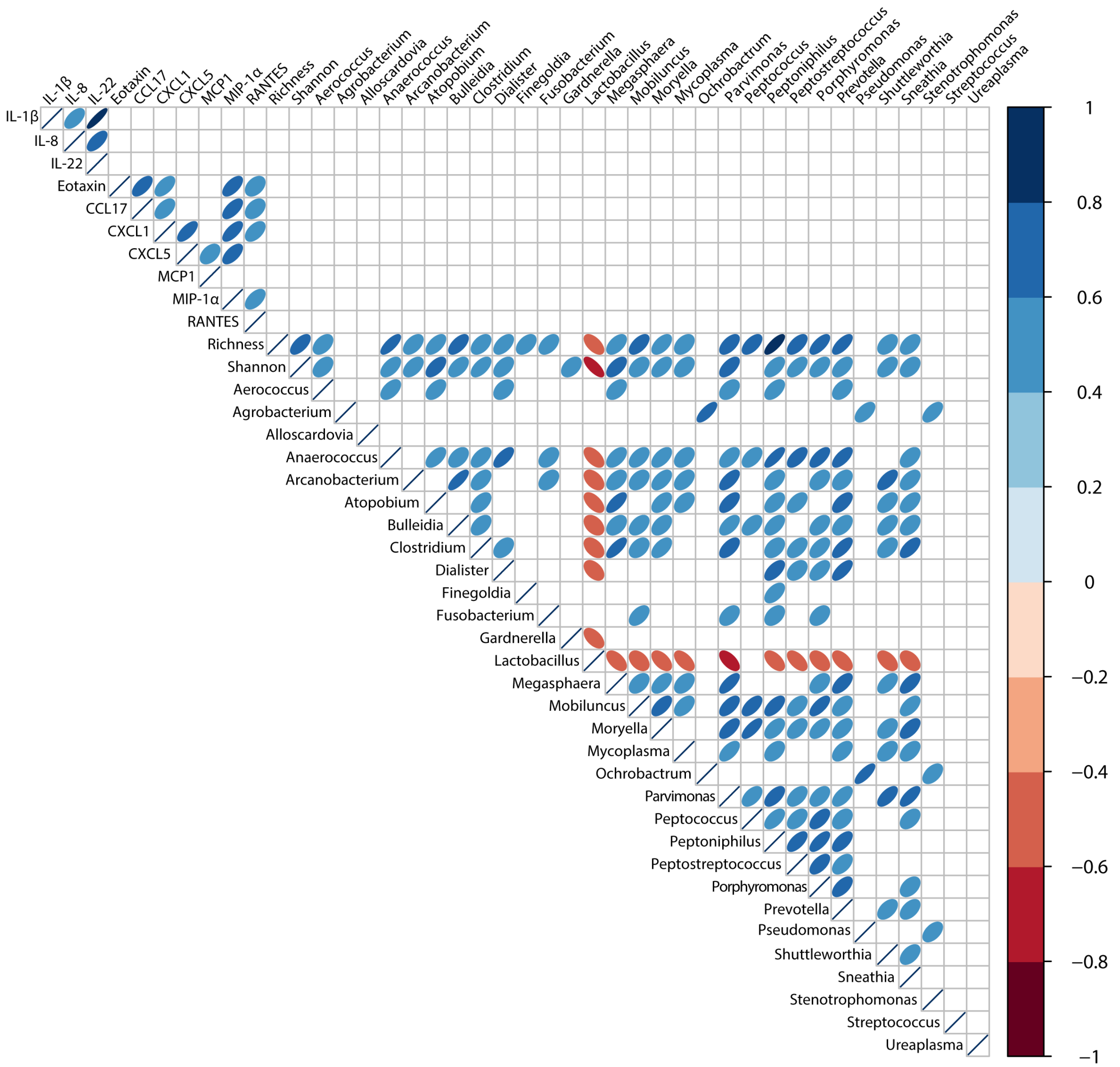

Supplementary Figure 3. Correlations between the vaginal microbiota and genital inflammation in women with symptomatic cervical ectopy. Correlations were computed using Spearman test, and visualized using corrplot. Only *p* values< 0.01 are shown. Positive rho values are shown in blue, negative rho values shown in red, the intensity of the color is proportional to the strength of the rho value.
