# Supplementary material for: Altered Vaginal Microbiota Composition Correlates With Human Papillomavirus and Mucosal Immune Responses in Women With Symptomatic Cervical Ectopy
Source: Front Cell Infect Microbiol. 2022 May 17;12:884272. doi: 10.3389/fcimb.2022.884272 (PMC9152460; doi:10.3389/fcimb.2022.884272)
Supplement: Supplementary file 4 [file Table_1.docx]

**Supplementary TABLE 1 |** Distribution of HPV genotypes in women with cervical ectopy and multiple HPV infection.

| HPV Types | No.  (N=26) |
| --- | --- |
| HPV 6, 11, 58 | 1 |
| HPV 6, 16, 59 | 2 |
| HPV 11, 16 | 1 |
| HPV 11, 18, 31 | 1 |
| HPV 11, 31 | 1 |
| HPV 11, 39 | 1 |
| HPV 16, 18 | 2 |
| HPV 16, 18, 31 | 1 |
| HPV 16, 31, 45, 53, 59 | 1 |
| HPV 16, 31, 45, 58 | 1 |
| HPV 16, 31, 59 | 1 |
| HPV 16, 39 | 1 |
| HPV 16, 53 | 1 |
| HPV 16, 58, 66 | 1 |
| HPV 16, 66 | 1 |
| HPV 18, 6 | 1 |
| HPV 18, 39, 53 | 1 |
| HPV 26, 39 | 1 |
| HPV 31, 35, 39, 53, 56, 59, 68, 73 | 1 |
| HPV 31, 39 | 1 |
| HPV 31, 52, 53 | 1 |
| HPV 45, 51, 52, 53, 58, 68 | 1 |
| HPV 51, 53, 58, 66 | 1 |
| HPV 52, 58 | 1 |

HPV, Human Papillomavirus
