# Supplementary material for: Altered Vaginal Microbiota Composition Correlates With Human Papillomavirus and Mucosal Immune Responses in Women With Symptomatic Cervical Ectopy
Source: Front Cell Infect Microbiol. 2022 May 17;12:884272. doi: 10.3389/fcimb.2022.884272 (PMC9152460; doi:10.3389/fcimb.2022.884272)
Supplement: Supplementary file 5 [file Table_2.docx]

**Supplementary TABLE 2** | Clinical, demographic and risk factors associated with HPV infection of the sub-cohort of women for assessment of genital inflammation and vaginal microbiota by 16S sequencing.

| **Characteristic** | **HPV-pos (N=35)** | **HPV-neg (N=28)** | ***p* value** |
| --- | --- | --- | --- |
| Age (years) | 30.09 | 33.36 | 0.0913^a^ |
| Parity | 2.14 | 2.21 | 0.6066^a^ |
| Menarche (years) | 12.94 | 12.43 | 0.1010^a^ |
| Age of sexual debut (years) | 17.20 | 17.79 | 0.5353^a^ |
| Number of sexual partners | 2.37 | 1.61 | 0.0163^a^* |
| Smoking status (%)  Current smoker  Non-smoker | 4 (11.4)  31 (88.6) | 5 (14.3)  23 (85.7) | 0.4687^b^ |
| Contraception (%)  None  Physical^c^  Hormonal^d^  Chirurgical^e^ | 13 (37.1)  12 (34.3)  5 (14.3)  5 (14.3) | 11 (39.3)  9 (32.1)  5 (17.9)  3 (10.7) | 0.9559^b^ |
| HPV vaccine (%)  Yes  No | 6 (17.1)  29 (82.9) | 3 (10.7)  25 (89.3) | 0. 5250^b^ |
| Prior pap smear (%)  Yes  No | 20 (57.1)  15 (42.9) | 18 (64.3)  10 (35.7) | 0.5647^b^ |
| Chronic diseases and conditions (%)  Asthma  Diabetes  Hypertension  Colitis | 1 (2.8)  1 (2.8)  1 (2.8)  1 (2.8) | 0 (0.0)  1 (3.6)  1 (3.6)  0 (0.0) | 0.5758^b^ |

Values are given as mean and number (%).

HPV, Human Papillomavirus

HPV-pos, HPV positive

HPV-neg, HPV negative.

^a^By Mann-Whitney test

^b^By Chi-square test

^c^Includes condoms, and intrauterine device (IUD)

^d^Includes implants, injections, patches, and pills

^e^Tubal ligation surgery

**p* < 0.05 was considered significant
