# Supplementary material for: Altered Vaginal Microbiota Composition Correlates With Human Papillomavirus and Mucosal Immune Responses in Women With Symptomatic Cervical Ectopy
Source: Front Cell Infect Microbiol. 2022 May 17;12:884272. doi: 10.3389/fcimb.2022.884272 (PMC9152460; doi:10.3389/fcimb.2022.884272)
Supplement: Supplementary file 6 [file Table_3.docx]

**Supplementary TABLE 3 |** HPV prevalence and genotype distribution in the sub-cohort of women for assessment of genital inflammation and vaginal microbiota by 16S sequencing (N=63).

| **HPV Genotypes** | **n/N** | **%** |
| --- | --- | --- |
| Negative | 28/63 | 44.4 |
| Any HPV type | 35/63 | 55.6 |
| Single HPV infection  HPV16  HPV18  HPV31 | 20/35  8  5  7 | 57.1 |
| Multiple HPV infection  HPV16, 18  HPV16, 39  HPV16, 53  HPV16, 11  HPV18, 6  HPV16, 31, 59  HPV16, 58, 66  HPV16, 6, 59  HPV18, 11, 31  HPV18, 39, 53  HPV31, 52, 53  HPV16, 31, 45, 58  HPV31, 35, 39, 56, 59 | 15/35  2  1  1  1  1  1  1  2  1  1  1  1  1 | 42.9 |

HPV, Human Papillomavirus

.
