# Supplementary material for: Altered Vaginal Microbiota Composition Correlates With Human Papillomavirus and Mucosal Immune Responses in Women With Symptomatic Cervical Ectopy
Source: Front Cell Infect Microbiol. 2022 May 17;12:884272. doi: 10.3389/fcimb.2022.884272 (PMC9152460; doi:10.3389/fcimb.2022.884272)
Supplement: Supplementary file 7 [file Table_4.docx]

**Supplementary TABLE 4** | Samples showing levels of cytokines and chemokines below the lower level of detection (LLOD)

| **Analyte** | **HPV-pos**  **≤LLOD, N (%)** | **HPV-neg**  **≤LLOD, N (%)** |
| --- | --- | --- |
| Cytokines |  |  |
| IL-1β | 2 (5.71) | 0 (0.00) |
| IL-2 | 26 (74.29) | 24 (85.71) |
| IL-4 | 29 (82.86) | 24 (85.71) |
| IL-5 | 35 (100.00) | 28 (100.00) |
| IL-6 | 14 (40.00) | 10 (35.71) |
| IL-8 | 0 (0.00) | 0 (0.00) |
| IL-9 | 26 (74.29) | 21 (75.00) |
| IL-10 | 35 (100.00) | 27 (96.43) |
| IL-12p | 33 (94.29) | 23 (82.14) |
| IL-13 | 31 (88.57) | 25 (89.29) |
| IL-17A | 35 (100.00) | 28 (100.00) |
| IL-17B | 32 (91.43) | 26 (92.86) |
| IL-21 | 17 (48.57) | 14 (50.00) |
| IL-22 | 4 (11.43) | 0 (0.00) |
| TNF-α | 31 (88.57) | 23 (82.14) |
| IFN-γ | 23 (65.71) | 12 (42.86) |
| Chemokines |  |  |
| IP-10 | 13 (37.14) | 12 (42.86) |
| Eotaxin | 10 (28.57) | 6 (21.43) |
| CCL17 | 0 (0.00) | 0 (0.00) |
| MCP-1 | 7 (20.00) | 3 (10.71) |
| RANTES | 6 (17.14) | 6 (21.43) |
| MIP-1α | 0 (0.00) | 0 (0.00) |
| MIG | 13 (37.14) | 9 (32.14) |
| CXCL5 | 5 (14.29) | 3 (10.71) |
| MIP-3α | 15 (42.86) | 9 (32.14) |
| CXCL1 | 3 (8.57) | 4 (14.29) |
| I-TAC | 23 (65.71) | 17 (60.71) |
| MIP-1β | 15 (42.86) | 8 (28.57) |

Values are given as mean.

HPV, Human Papillomavirus

HPV-pos, HPV positive samples

HPV-neg, HPV negative samples

.
